# An Anonymous Authentication and Key Update Mechanism for IoT Devices Based on EnOcean Protocol

**DOI:** 10.3390/s22176713

**Published:** 2022-09-05

**Authors:** Yi Wu, Tao Feng

**Affiliations:** School of Computer and Communication, Lanzhou University of Technology, Lanzhou 730050, China

**Keywords:** smart lighting control, EnOcean protocol, colored Petri net, Dolev–Yao attacker model

## Abstract

EnOcean, a commonly used control protocol in smart lighting systems, provides authentication, as well as message integrity verification services, and can resist replay attack and tamper attack. However, since the device identity information transmitted between sensors in smart lighting control systems is easily accessible by malicious attackers, attackers can analyze users’ habits based on the intercepted information. This paper analyzed the security of the EnOcean protocol using a formal analysis method based on the colored Petri net (CPN) theory and the Dolev–Yao attacker model and found that the protocol did not anonymize the device identity information and did not have a communication key update mechanism, so an attacker could easily initiate a key compromise impersonation attack (KCIA) after breaking the pre-shared communication key. To address the above security issues, this paper proposed an EnOcean-A protocol with higher security based on the EnOcean protocol. The EnOcean-A protocol introduced a trusted third-party server to send communication keys to communication devices because devices must obtain different communication keys from the trusted third-party server each time they communicated. Thus, this protocol could resist a KCIA and achieve forward security. Meanwhile, the device identity information was anonymized using a homomorphic hash function in the EnOcean-A protocol, and the dynamic update mechanism of the device identity information was added so that an attacker could not obtain the real identity information of the device. Finally, the formal analysis of the EnOcean-A protocol showed that the new protocol could resist a KCIA and ensure the anonymity and untraceability of the communication device, which had higher security compared with the EnOcean protocol.

## 1. Introduction

The smart lighting control system, a novel internet of things (IoT) product, has emerged quickly in the industrial and commercial markets due to the rapid development of wireless communication technology and sensor technology [1]. Not only can smart lighting control systems offer lighting for industrial environments, but they can also give homeowners more intelligent living spaces. For instance, a lighting control system can activate a fixed-position lighting device in accordance with a user’s location when the user opens a door and enters a room [2].

The application process for the smart lighting control system must gather data about users’ everyday lives, including their location and entry and leave times, in order to properly perform. Private information about users’ movements and positions is gathered by sensing devices to offer more intelligent service experiences [3,4]. However, the majority of these sensor devices communicate data over wireless networks. Wireless transmission channels’ openness makes it simple for malicious attackers to intercept and tamper with the sent data. In order to determine if a smart lighting control system can guarantee security, it is crucial to ensure that personal data is not compromised throughout the IoT application process. Neither the current nor the updated lighting control protocol can, however, provide a greater level of data privacy protection.

One of the protocols used to ensure secure communication for IoT devices is Zigbee [5]. Farha et al. [6] proposed an enhanced ZigBee protocol scheme employing an enhanced timestamp mechanism that is provided because the security issue that the ZigBee protocol is vulnerable to is replay attack. Khanji et al. [7] suggested updating the key management and key update mechanisms to address the security issues with the ZigBee protocol, including denial of service (DOS), flooding, desynchronization, and wormhole attacks. In smart lighting control systems, the Bluetooth protocol is frequently employed [8,9]. Lonzetta et al. [10] and Zhang et al. [11] analyzed smart home devices employing the Bluetooth protocol that were vulnerable to man-in-the-middle (MITM) attacks, wormhole attacks, and sniffer attacks. Long-range wide-area network (LoRaWANs) are often used in lighting control systems due to their low power consumption and capacity for long-distance communication [12,13,14]. A new key management protocol has been proposed in [15,16,17,18,19] to address the issues of key management and key update in LoRaWAN protocol and to fight against key compromise and replay attack. Nutun et al. [20] discovered security issues in the LoRaWAN protocol, such as synchronous attack and MITM attack, and proposed security approaches to improve the protocol’s security. Tsai et al. [21] suggested a novel session key generation approach by integrating elliptic curve cryptography (ECC) with an advanced encryption standard (AES) encryption method to generate a secure session key between devices in order to address the security flaws between application layers in the LoRaWAN protocol. According to the aforementioned paper, existing and enhanced smart lighting control protocols primarily offer security protection against MITM attacks, DOS attacks, and replay attacks but do not offer measures to anonymize sensor device information.

EnOcean Alliance proposed the EnOcean wireless transmission system [22]. In March 2012, the International Electrotechnical Commission (IEC) approved the EnOcean wireless communication standard as an international standard (ISO/IEC 14543-3-10). EnOcean is a wireless communication system with low power requirements, ad hoc networking, strong security, and reliability. All sensors, wireless switches, and other equipment that support the EnOcean protocol do not require batteries or other power sources. This reduces the need for complex equipment maintenance in the future, especially for smart lighting control systems [23].

Since smart lighting control systems are widely used in life, providing people with a more convenient and smarter life experience, most of the existing intelligent lighting control systems are able to provide message encryption, identity authentication, and message integrity verification services. However, because the device identity information transmitted between sensors in an intelligent lighting control system is easily accessible by malicious attackers, attackers can analyze a user’s living habits based on the intercepted information. Through the related literature, it can be found that most of the existing smart lighting control protocols cannot anonymize the device identity, so this paper proposes a protocol with higher security based on the EnOcean protocol. The main contributions of this paper are as follows:

- In this paper, we use CPN theory and the Dolev–Yao attacker model to formally analyze the EnOcean protocol. Through a security analysis, we find that the EnOcean protocol is able to provide authentication and message integrity verification services, but the EnOcean protocol does not anonymize the identity information of the communication devices, so attackers can analyze a user’s living habits based on the eavesdropped device identity information. The formal analysis reveals that the EnOcean protocol cannot resist a KCIA.
- To address the above security issues, this paper proposes the EnOcean-A protocol with higher security. The EnOcean-A protocol introduces a trusted third-party server to distribute communication keys to communication devices, and the devices can obtain new communication keys from the trusted third-party server before communication, which can resist KCIA and provide forward security. The EnOcean-A protocol uses a homomorphic hash function to anonymize the device identity information and adds a dynamic update mechanism for device identity information so that an attacker cannot obtain the real identity information of the device, thus providing unlinkability of the device identity information.
- Finally, the security of EnOcean-A protocol is analyzed by a formal analysis method, and it is found that the EnOcean-A protocol has higher security compared with the EnOcean protocol.

## 2. Related Works

Several distinct anonymous approaches have been proposed for IoT devices. The traceability of device identities in smart home systems enables attackers to track device activity and infer users’ daily routines. Sadri et al. [24] proposed a solution that uses two-factor authentication to authenticate user identification and symmetric key encryption to transmit data. Banerjee et al. [25] presented a simple, anonymous authentication mechanism using the user’s smart card, password, and unique biometric data. Fakroon et al. [26] proposed a unique user authentication mechanism that utilized physical information. User information must be saved on the device for the three physical-factor-based authentication mechanisms described above. They are not suitable for protocols used only for authentication between devices. Without a trusted third-party server involved, Hajian et al. [27] proposed a key agreement technique for mutual authentication between two devices. In this protocol, the registration and key update phases were conducted in a common channel, in addition to the identity authentication phase. Li et al. [28] suggested an anonymous authentication and key agreement mechanism (AAKA). To accomplish authentication and privacy protection, this protocol made use of dynamic sequence numbers and dynamic random numbers. The proposed protocol worked well for identity authentication between a user, sensor node, and gateway and did not rely on a trusted third-party server. Lightweight, anonymous protocols cannot be developed without a third-party server since the computing capabilities of lighting sensor devices do not allow them to calculate keys and complicated encryption algorithms. A lightweight, secure smart home protocol based on mutual anonymous authentication and device key agreement was proposed in Hasan et al. [29] and Banerjee et al. [30]. However, only the user, sensor, and gateway nodes’ identities could be authenticated using this type of approach. Rasheed et al. [31] presented a zero-knowledge proof-based authentication mechanism. However, this approach could be used with a multicast environment’s identity authentication protocol. It was not necessary to authenticate between two nodes. Shuai et al. [32] proposed an effective anonymous authentication method based on ECC suggested to be employed in smart home environments. To resist against replay attack and prevent clock synchronization, the scheme employed random numbers. This method utilized the ECC public key encryption standard, which has certain performance requirements for devices. An anonymous security framework (ASF) appropriate for a smart home environment was suggested in Kumar et al. [33]. This framework provided device anonymity and effective authentication and key agreement scheme. The device identity in this method, however, was static and could not be utilized to create untraceable communication devices. Table 1 provides a comparative analysis of the above articles.

Hofer-Schmitz [34] presented the first formal analysis method for the EnOcean protocol and discovered design flaws in the EnOcean protocol through the formal analysis method. Based on EnOcean’s security documentation, the authors used the pi calculus formal analysis method and the ProVerif tool to test the security of the EnOcean protocol. The results showed that, although the EnOcean protocol could guarantee its stated security, it did not encrypt the identity information of the sending device during the teach-in phase, so an attacker could send an intercepted message to another receiving device. The author proposed a solution to the above problem. However, this formal analysis did not reveal that the EnOcean protocol was insecure against KCIA and that the protocol did not anonymize the identity information of the device during operation.

The literature mentioned above revealed that the majority of the current anonymous authentication mechanisms and schemes have the following issues: (a) The IoT terminal devices may not have enough computing power to support necessary activities if they rely on ECC public key encryption. (b) While ignoring the anonymous authentication between sensors, they focus primarily on the three-party authentication between the user, the sensor, and the gateway. (c) Since there is no method for dynamic identification information modification and only device information may be anonymized, sensor device untraceability is not achievable. Moreover, the existing security analysis of the EnOcean protocol did not suggest that there was no device anonymization operation in the EnOcean protocol. Under the condition that the sensor nodes are limited in power energy, communication capability, and computational and storage capacity of the chip, this paper introduces a trusted third-party server to send communication keys to the communication nodes and provides anonymization for devices using only homomorphic hash functions. 

## 3. Preliminary Knowledge

### 3.1. EnOcean Protocol

The EnOcean protocol includes two phases: teach-in and authentication [34]. For wireless network authentication, a teach-in phase must be performed so the receiver can determine whose device sent the message. The EnOcean protocol employs a 32-bit chip ID as the device identification [35]. Devices are able to determine if a message needs to be processed based on the sender ID learned during the teach-in phase. The pre-shared key is typically written on a sticker on the sender module. EnOcean uses variable AES encryption and cipher-based message authentication codes to verify message integrity.

The EnOcean protocol is shown below, and Table 2 shows specific symbols.

Step 1: A and B switch on the learn mode. A generates the RLC and Key  and encrypts them using the PSK. A transmits $ID_{A}$, $ID_{B}$, and the encrypted data to B. B confirms whether the message is received after receiving it. B discards the message if it has already been received. Otherwise, the teach-in phase is over, and the Key and RLC are saved.

Step 2: For the authentication phase, A sends a challenge message $\left(ID_{A},\;ID_{B}\right)$ to B.

Step 3: After receiving the message, B generates an RND and sends $\left(ID_{A},ID_{B},RND\right)$ to A.

Step 4: A calculates the CMAC value of the Payload and RND using the Key after receiving the message. The Payload and CMAC are encrypted with the Key. $ID_{A}$, $ID_{B}$, and the encrypted data are sent from A to B.

**Figure 1:**
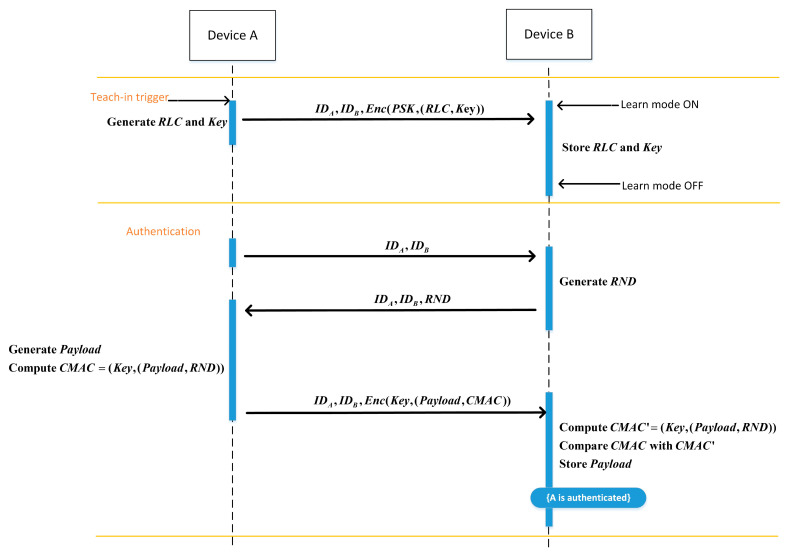
EnOcean protocol data flow.

Step 5: B receives the message and uses the Key to decrypt it in order to acquire the Payload and CMAC. B also calculates the CMAC’ using the Payload and RND. A is successfully authenticated, and B saves the Payload if the CMAC=CMAC’. In Figure 1, the specific procedure is displayed.

### 3.2. Homomorphic Hash Function

Homomorphic hash function refers to a hash algorithm with homomorphism [36,37]. If $Z_{p}$ is a finite field of positive integers p and the vector g is a 1×l row vector, then each entry is a member of $Z_{p}$ of order q, and l is a positive integer. The elements $m_{i},\;m_{j}$, $i,j\in \left\{1,2,\ldots ,n\right\}$ in the data block $F=\left\{m_{1},\;m_{2},\ldots ,m_{n}\right\}$ are all elements of the vector $Z_{p}$, and $H\left(\cdot \right)$ is homomorphic hash function, while t is a positive integer:

$$H\left(m_{i}\right)=\overset{l}{\underset{t=1}{\prod }}g_{t}^{m_{t,i}}\mathrm{mod}\;p\;$$

Then, the hash of the addition of two vectors $m_{i}$ and $m_{j}$ is as follows:

$$H\left(m_{i}+m_{j}\right)=\overset{l}{\underset{t=1}{\prod }}g_{t}^{m_{t,i}{+m}_{t,j}}\;\mathrm{mod}\;p=\overset{l}{\underset{t=1}{\prod }}g_{t}^{m_{t,i}}g_{t}^{m_{t,j}}\;\mathrm{mod}\;p=\overset{l}{\underset{t=1}{\prod }}g_{t}^{m_{t,i}}\mathrm{mod}\;p\;\times \overset{l}{\underset{t=1}{\prod }}g_{t}^{m_{t,j}}\mathrm{mod}\;p=H\left(m_{i}\right)\times H\left(m_{j}\right)\;$$

It can be seen that the hash algorithm is homomorphic. Homomorphic hash functions also have a collision avoidance property. For a homomorphic hash function, for any two pieces of information $m_{1}$, $m_{2}$, and for real numbers $\;w_{1}$, $w_{2}$, the homomorphism has the following:

$${H(w}_{1}m_{1}{+w}_{2}m_{2}{)=H(m}_{1}{)}^{w_{1}}{H(m}_{2}{)}^{w_{2}}\;$$

The attacker does not exist in the probability polynomial algorithm, which can obtain $\left(m_{1},m_{2},m_{3},w_{1},w_{2}\right)$, where $m_{3}\neq w_{1}m_{1}+w_{1}m_{2}$, and $m_{3}$ is a piece of information:

$$H\left(m_{3}\right){=H(m}_{1}{)}^{w_{1}}{+H(m}_{2}{)}^{w_{2}}$$

### 3.3. CPN Modeling Tool

CPN is a modeling language [38,39] that successfully combines the standard meta language (ML) for net systems with the Petri net theory. Common systems can be handled by CPN, and it can simulate and analyze concurrent systems. A CPN model of a system is typically executable and can describe both the current state of the system and efforts to change it. This event is a transition in the CPN model. CPN Tools is essential in dealing with systems such as concurrency, interactivity, and synchrony due to its advantages in modeling and verifying model systems.

### 3.4. Dolev–Yao Attacker Model

The Dolev–Yao attacker model proposed by D. Dolev and A. Yao [40] formally characterized the behavior of an attacker and offered a mathematical model for testing public key cryptography protocol. Researchers can benefit by focusing on the inherent security qualities of protocols rather than the security of cryptographic algorithms by discussing the security properties of protocols based on the assumption that the cryptosystem is “perfect”.

The attacker has the following functions:

- The attacker has access to the open channel. The data transmitted between entities while the protocol is executing can be intercepted, altered, and replayed by the attacker;
- The attacker can encrypt, decrypt, split, and combine the original message through the known content to forge the message content;
- Using the identity of the sensor that was eavesdropped, the attacker can determine whether it was delivered by the same device;
- The attacker can break the long-used key and simulate the device to launch an attack using this key.

## 4. EnOcean Protocol HCPN Modeling

### 4.1. EnOcean Protocol Color Set Definition

The color set is established for the four messages exchanged between the receiver and the sender. First of all, communication information is composed of four pieces of meta-information: ID, RANDOM, KEY, and PAYLOAD. ID indicates the identity of the device chip, RANDOM indicates a random number, PAYLOAD indicates the payload sent by the sender, and KEY indicates the encryption key. The MSG1 type indicates that teach-in messages are sent from the sender to the receiver. The MSG2 type indicates that the sender sends an identity authentication request to the receiver. The MSG3 type indicates that the receiver sends a random number for authentication to the sender. The MSG4 type indicates that the sender sends the authentication calculation results to the receiver. The specific color set definitions are shown in Table 3.

### 4.2. Formal Analysis of EnOcean Protocol

In this study, a top-down formal analysis of the EnOcean protocol was modeled using CPN Tools. The protocol submodule was established after the protocol top-level model was defined. The transition was represented by a rectangle, the place was represented by an ellipse, and the substitution transition was represented by a double-line rectangular transition, indicating that the transition had more detailed submodels.

**Figure 2:**
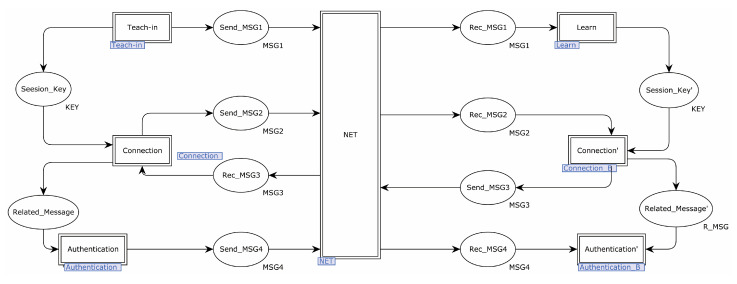
EnOcean protocol top-level model.

The EnOcean protocol’s top model had 12 places and 7 substitution transitions. The substitution transition of Teach-in represented the unique procedure of the sender providing teach-in information to the receiver. The substitution transition of Learn represented how teach-in signals were received and analyzed by the receiver. The substitution transition of Connection represented the procedure through which the sender sent an authentication request and received a random number. The substitution transition of Connection represented the procedure where the receiver received the authentication request and delivered a random number to the sender. The substitution transition of Authentication represented the transmission of the CMAC value to the receiver after it was calculated by the sender. The substitution transition of Authentication’ represented the procedure through which the receiver compared the CMAC values supplied by the sender. Figure 2 illustrates the specifics.

**Figure 3:**
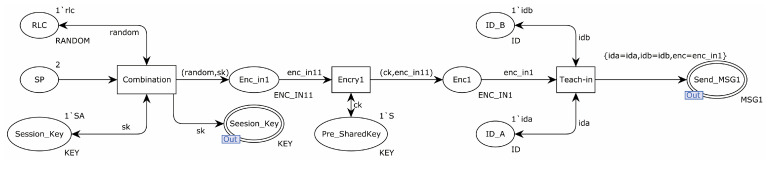
Substitution transition teach-in internal model.

A detailed explanation of the partial substitution transition follows. The sub-model that replaced the Teach-in transition is shown in Figure 3. The rlc and session key were first sent by the transition of Combination to the transition of Encry1, which then encrypted the message using the pre-shared key and transmitted it to the transition of Teach-in. Together with the sender and receiver’s identities, the transition of Teach-in delivered the encrypted message to the place Send_MSG1.

**Figure 4:**
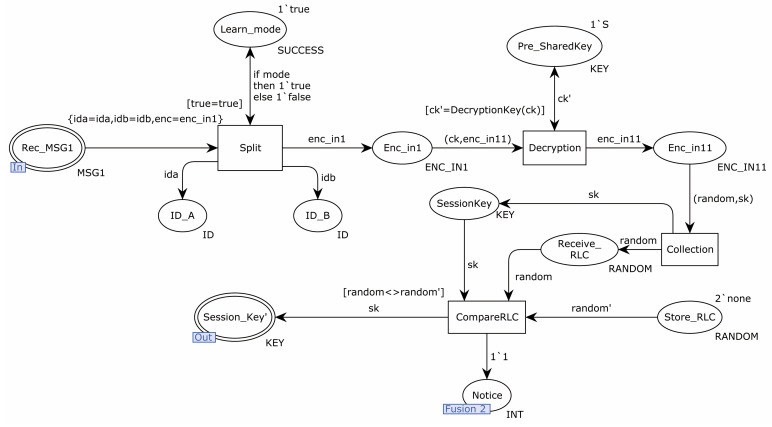
Substitution transition learn internal model.

Figure 4 is the internal model of the substitution transition Learn. First, the transition of Split split the received MSG1 information, stored the identity information of the sender and receiver in places ID_A and ID_B, and sent the remaining information to the transition of Decryption. The transition of Decryption used the pre-shared key to decrypt the received message. If the key was different, the decryption failed. Otherwise, the decryption message was sent to the transition of Collection. The transition of Collection sent the RLC in the decryption message to the place Store_RLC for saving and the session key to the place Session_Key′.

**Figure 5:**
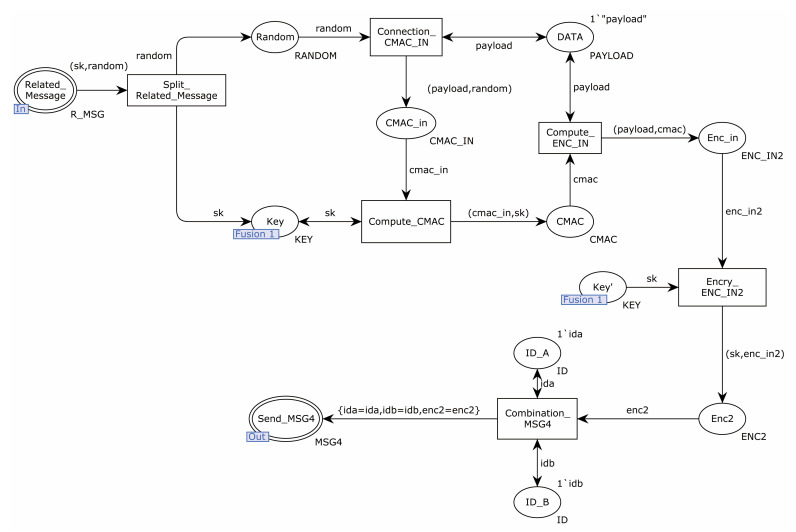
Substitution transition authentication internal model.

Figure 5 shows the internal model of the substitution transition of Authentication. The place Related_Message sent a message to the transition of Split_Related_Message. The transition of Split_Related_Message split the received message and sent the random number to the transition of Connection_CMAC_IN and the session key to the transition of Compute_CMAC. The transition of Connection_CMAC_IN combined random numbers and authentication messages to the transition of Compute_CMAC. The transition of Compute_CMAC computed the CMAC value of the received message using the session key and sent the CMAC to the transition of Compute_ENC_IN. The transition of Encry_ENC_IN2 encrypted the received message using the session key and sent the encrypted message to the transition of Combination_MSG4. The transition of Combination_MSG4 sent the received encrypted message and sender and receiver identities to the place Send_MSG4.

**Figure 6:**
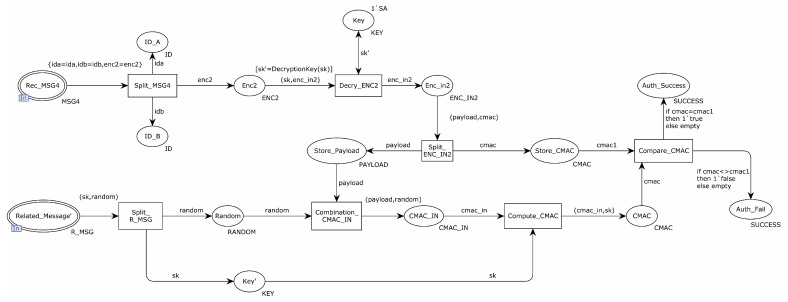
Substitution transition authentication internal model.

Figure 6 shows the internal model of the substitution transition of Authentication. The transition of Split_MSG4 split the received message, saved the identity of the sender and receiver to places ID_A and ID_B, and sent the encrypted message to the transition of Decry_ENC2. The transition of Decry_ENC2 used the session key to decrypt the encrypted message. If the encrypted message was not encrypted with the session key, the decryption failed. Otherwise, the decryption message was sent to the transition of Split_ENC_IN2. The transition of Split_ENC_IN2 split and decrypted the message, saved the CMAC value in place Store_CMAC, and saved the authentication message in place Store_Payload. Place Related_Message’ sent a message to place Split_R_MSG. Place Split_R_MSG split the received message and sent the random number to the transition of Combination_CMAC_IN. The transition of Combination_CMAC_IN sent random numbers and authentication messages to the transition of Compute_CMAC, which computed the CMAC value using the session key and sent the result to the transition of Compare_CMAC. The transition of Compare_CMAC compared the received CMAC value with the calculated CMAC value. If the values were equal, it indicated that the receiver authenticated the sender successfully and saved the authentication success message to the place Auth_Success. Otherwise, the authentication failure message was saved to the place Auth_Fail.

### 4.3. EnOcean Protocol Consistency Analysis

State-space analysis methods were used to analyze the EnOcean protocol’s conformance. The numbers of nodes and arcs in a state-space were the same as the numbers of strongly connected nodes and strongly connected arcs, according to an analysis of the state-space results in Table 4. This finding shows that the established EnOcean protocol model did not result in state cycles and that all the state nodes were reachable. One dead node indicated that the receiver handled all the requests, allowing the model to function as intended.

### 4.4. EnOcean Protocol Security Evaluation Based on Dolev–Yao Attacker Model

**Figure 7:**
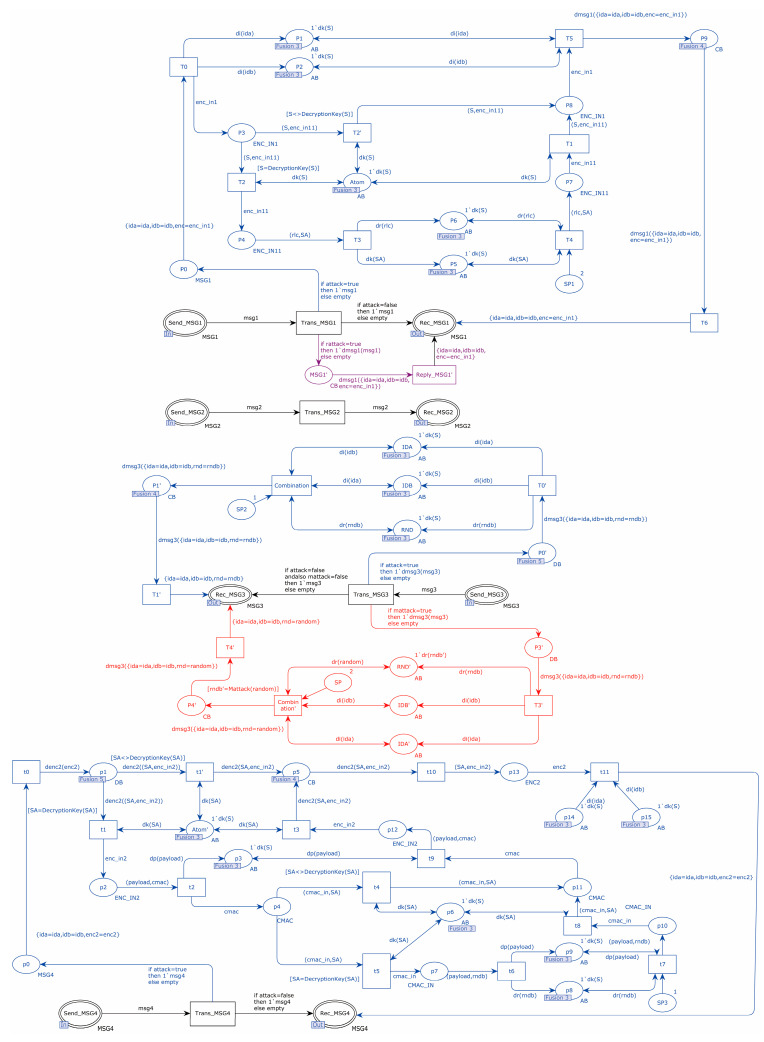
EnOcean protocol attacker model.

The replay attack, tamper attack, and KCIA were introduced in the network transport layer of the EnOcean protocol model. The attacker could analyze whether the information was sent by the same device based on the intercepted device identity information, and when the attacker obtained the pre-shared key, he could decrypt the information transmitted between devices for a second time and tamper with the transmitted information. The places and transitions marked in blue in Figure 7 simulate a KCIA; the transitions T0 and t0 intercept the information during the protocol transmission; the places P1 and P3 are able to store the decomposed and to-be-decomposed information; and the places P1, P2, Atom, P6, P5, IDA, IDB, RND, p3, p8, p9, and Atom store the atomic information. Transitions T2, T2′, t1, and t1′ indicate the decryption of intercepted messages with a known key. Transitions T5 and t11 indicate the reassembly of the decomposed messages. The red part in Figure 7 indicates that a tamper attack was simulated, and the tamper attack is launched by transition T3′. The purple part in Figure 7 indicates that a replay attack is simulated, and the replay attack is launched through the transition of Reply_MSG1′.

### 4.5. EnOcean Protocol Security Attribute Verification Analysis

All state nodes in the attacker model of this protocol were reachable, as shown by the state space report on the attacker model in Table 5. The numbers of state space nodes and directed arcs were equal to the numbers of strongly connected nodes and strongly connected arcs.

**Figure 8:**
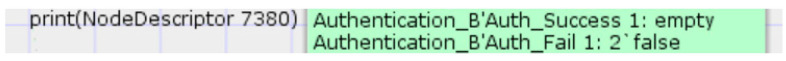
Identity authentication result on the receiver.

**Figure 9:**
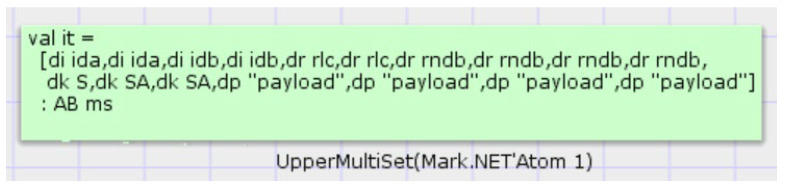
Attacker obtains information query results.

As indicated in Table 5, an analysis of the state-space data from the EnOcean protocol added to the attacks. A tamper attack was implemented in the protocol’s transport layer of MSG3. Given that MSG3 messages were sent in plaintext, an attacker could directly alter random integers in the message. However, the CMAC algorithm was employed by this protocol. The CMAC value was determined by the sender using random numbers and authentication messages, and the matching CMAC value was determined by the receiver using the same CMAC algorithm. The receiver concluded that the identity authentication of the sender failed when it compared the CMAC values and discovered that the two CMAC values were different. The query statement found the failed value in the place Auth_Fail on the receiver, indicating that identity authentication failed. Figure 8 displays the details. In MSG1, a replay attack was added at the transport layer. When an attacker launched a replay attack, the receiver checked to see whether the RLC in the message was stored in the MSG1 messages. The receiver recognized that a replay attack had taken place if the RLC was already present and stopped the further actions. Replay attacks led to the 23 dead transitions in the state-space report. The KCIA was used at the transport layer since the attacker already knew the pre-shared key used by the devices, enabling the attacker to decrypt MSG1. The attacker could access the session key after decrypting MSG1. The attacker could successfully acquire the random number and CMAC value throughout the subsequent communication procedure using the session key. Using the query statement, Figure 9 represents the secret information obtained by the attacker after launching a KCIA. Moreover, it can be found in the attacker’s places that the sender and the receiver used the same identity ID during the two communications, so the attacker could evaluate the user’s life based on the device identity information.

The EnOcean protocol state-space analysis revealed that the protocol could successfully resist replay attacks and tamper attacks. The EnOcean protocol, however, was unable to resist the KCIA. An attacker could successfully gain the session key used by the device during communication if the attacker broke the pre-shared key. As a result, the attacker had access to all the private information shared via the communication devices. This protocol did not ensure the anonymity of the communication devices since the sender and receiver’s identification did not change during the two communication operations.

## 5. Device Anonymity and Key Update Scheme Based on EnOcean Protocol

### 5.1. EnOcean-A Protocol

In order to address that the communication device was not anonymized in the EnOcean protocol and was insecure against a KCIA, this paper proposed the EnOcean-A protocol. Under the premise of low performance of chip and memory, we introduced a trusted third-party server to send communication keys to communication devices, and communication devices must obtain communication keys from the trusted third-party server every time they communicated. Moreover, the homomorphic hash function was used to anonymize the device identity information, and the dynamic update mechanism of the device identity information was introduced. In the EnOcean-A protocol, when the communication device started the teach-in process, first the sender initiated a communication key application request to the trusted third-party server, and the trusted third-party server, after verifying the identities of the sender and the receiver, sent the anonymous identity information and communication key of the receiver to the sender, sent the anonymous identity information and communication key of the sender to the receiver, and the trusted third-party server updated the hash of the device information. When the sender and the receiver received the identity information and communication key from the trusted third-party server, they first updated the identity information hash of their own device, and then the communicating parties started the teach-in process and authentication process based on the original EnOcean protocol.

### 5.2. EnOcean-A Protocol Communication Process

**Figure 10:**
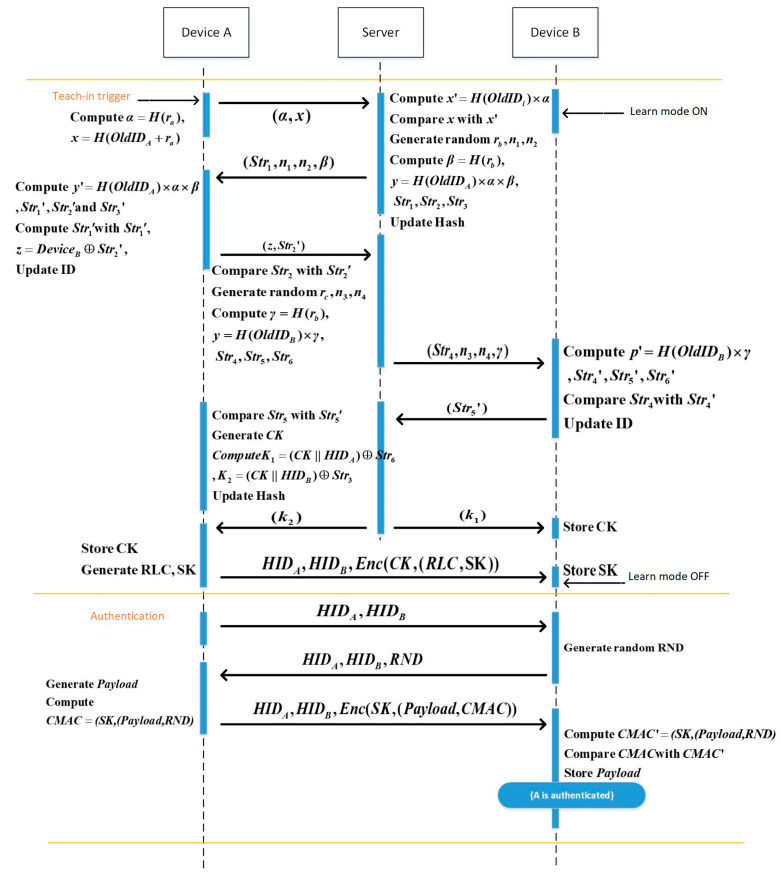
EnOcean-A protocol data flow.

The specific communication process of the EnOcean-A protocol is shown below. Table 6 lists the important symbols, and Figure 10 represents the specific communication process of the EnOcean-A protocol.

- Teach-in phase:

Step 1: When A sends a request for authentication to S, A picks a random number $r_{a}$ and computes $\alpha =H\left(r_{a}\right)$ and $x=H\left(OldID_{A}+r_{a}\right)$. A sends $\left(\alpha ,\;x\right)$ to S.

Step 2: After S receives $\left(\alpha ,\;x\right)$, S finds the stored user identification hash function group, calculates $x^{\prime }=H\left(OldID_{i}\right)\times \alpha$, and determines whether x=x’ exists. If it exists, then A authentication is successful; otherwise, authentication is not passed. After the authentication is passed, S selects a random number $r_{b}$ and calculates $\beta =H\left(r_{b}\right),\;y=H\left(OldID_{A}\right)\times \alpha \times \beta$. S generates two random numbers $n_{1}$ and $n_{2}$($0<n_{1},\;n_{2}<L,\;n_{1}\;\neq \;n_{2})$, where L is the length of y, and obtains three strings: $Str_{1}$, $Str_{2}$, and $Str_{3}$. $Str_{1}$ is the data y from the start position to position $n_{1}$, $Str_{2}$ is the data y from position $n_{2}$ to the end position, and $Str_{3}$ is the data y from $n_{1}$ to position $n_{2}$. S sends $\left(Str_{1},\;n_{1},\;n_{2},\;\beta \right)$ to A after the calculation is finished. At the same time, S needs to update the hash value inside the function group: $H\left(OldID_{A}\right)=H\left(NewID_{A}\right),\;H\left(NewID_{A}\right)=H\left(NewID_{A}\right)\times \alpha$.

Step 3: After A receives the message, $y‘=H\left(OldID_{A}\right)\times \alpha \times \beta$ is calculated, $Str_{1}^{’},\;\;Str_{2}’$, and $Str_{3}’$ are calculated by $n_{1}\;\mathrm{and}\;n_{2}$ and $Str_{1}$ is compared with $Str_{1}’$. If equal, it means A considers S authenticated successfully; otherwise, authentication fails. After successful authentication, A calculates $z=Device_{B}⊕Str_{2}’$, and A sends $\left(z,\;Str_{2}^{’}\right)$ to S. At the same time A updates the hash value of identity: $H\left(OldID_{A}\right)=H\left(NewID_{A}\right),\;H\left(NewID_{A}\right)=H\left(NewID_{A}+r_{a}\right)$.

Step 4: After receiving the data sent from A, S first compares $Str_{2}’$ with $Str_{2}$. If $Str_{2}^{’}=Str_{2}$, S considers A authenticated successfully and uses $Str_{2}$ to perform an XOR operation on the message z to obtain $Device_{B}$ and learns that A wants to communicate with B. S generates a random number $r_{c}$ and calculates $\gamma =H\left(r_{c}\right)$, $p=H\left(OldID_{B}\right)\times \gamma$. S generates two random numbers $n_{3}$ and $n_{4}$($0<n_{3},\;n_{4}<L,\;n_{3}\;\neq \;n_{4})$, where L is the length of p, to obtain $Str_{4}$, $Str_{5}$, and $Str_{6}$. $Str_{4}$ is the data p from the start position to position $n_{3}$, $Str_{5}$ is the data p from position $n_{4}$ to the end position, and $Str_{6}$ is the data from $n_{3}$ to position $n_{4}$. $\left(Str_{4},\;n_{3},\;n_{4},\;\gamma \right)$ is sent to B when the calculation is finished.

Step 5: After B receives the message $\left(Str_{4},\;n_{3},\;n_{4},\;\gamma \right)$, $p^{\prime }=H\left(OldID_{B}\right)\times \gamma$ is computed, and $Str_{4}^{’},\;\;Str_{5}’$, and $Str_{6}’$ are computed with $n_{3},\;n_{4}$. $Str_{4}$ and $Str_{4}’$ are compared. If they are equal, it means that B considers S authenticated successfully; otherwise, authentication fails. After successful authentication, B sends $Str_{5}’$ to S. At the same time, B updates the hash value of ID: H($OldID_{B})=H\left(NewID_{B}\right),\;H\left(NewID_{B}\right)=H\left(NewID_{B}\right)\times \gamma$.

Step 6: After S receives the message, $Str_{5}$ is compared with $Str_{5}’$. If equal, then S considers B authenticated successfully; otherwise, authentication fails. After successful authentication, S generates the communication key CK, calculates $K_{1}=\left(CK∥HID_{A}\right)⊕Str_{6}\;and\;K_{2}=\left(CK∥HID_{B}\right)⊕Str_{3}\;$, and finally sends $K_{1}$ to B and $K_{2}$ to A. At the same time, S needs to update the hash value: $H\left(OldID_{B}\right)=H\left(NewID_{B}\right),\;H\left(NewID_{B}\right)=H\left(NewID_{B}\right)\times \gamma$.

Step 7: After receiving the message, A uses $Str_{3}$ to perform an XOR operation on message $K_{2}$ to obtain the communication key CK and $HID_{B}$. After receiving the message, B uses $Str_{6}$ to perform an XOR operation on message $K_{1}$ to obtain the communication key CK and $HID_{A}$.

Step 8: A generates a rolling code RLC and a session key SK and then computes $M_{1}=E\left(CK,\left(RLC,\;SK\right)\right)$. A sends $\left(HID_{A},\;HID_{B},\;M_{1}\right)$ to B.

Step 9: After receiving the message, B decrypts $M_{1}$ with the communication key CK and obtains the rolling code RLC and the session key SK. The teach-in phase ends.

- Authentication phase:

Step 10: A sends $\left(HID_{A},\;HID_{B}\right)$ to B.

Step 11: After B receives the message, B generates a random number RND and sends $\left(HID_{A},\;HID_{B},\;RND\right)$ to A.

Step 12: After the message is received by A, if the verification is successful, A calculates $M_{2}=CMAC\left(SK,\;\left(p,\;RND\right)\right)$ and $M_{3}=E\left(SK,\;\left(p,\;M_{2}\right)\right)$ and sends $\left(HID_{A},\;HID_{B},\;M_{3}\right)$ to B.

Step 13: After B receives the message, B obtains the p and CMAC using the session key SK. B calculates $M_{2}’=CMAC\left(SK,\;\left(p,\;RND\right)\right)$, and if $M_{2}^{’}=M_{2}$, the data are confirmed to be complete, and B considers A authenticated successfully. The specific process is shown in Figure 10.

## 6. Formal Analysis of EnOcean-A Protocol

### 6.1. EnOcean-A Protocol HCPN Model

**Figure 11:**
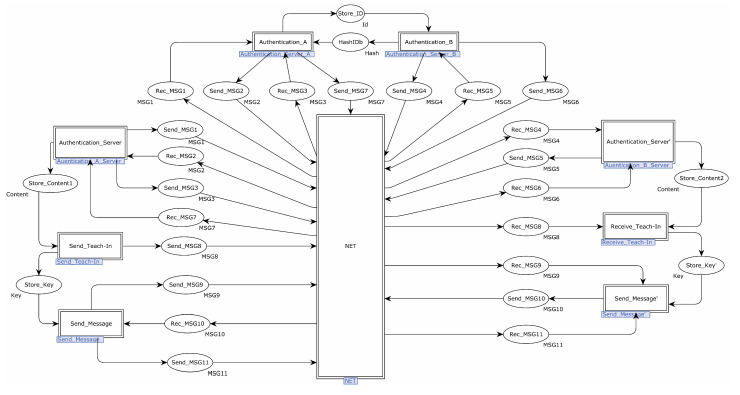
EnOcean-A top-level model.

The EnOcean-A protocol was modeled using CPN Tools. The EnOcean-A protocol’s top model had 28 places and 8 substitution transitions. The substitution transition Authentication_Server stood in for the process by which the sender made a communication key request to the trusted third-party server and acquired the communication key and the receiver’s identification hash value. The substitution transition Authentication_A stood in for the process where the trusted third-party server transmitted a communication key and the hash value of the receiver’s identity to the sender. The substitution transition Authentication_B represented the module that the trusted third-party server transmitted to the receiver for both the communication key and the hash value of the sender’s identity. The substitution transition Authentication_Server′ represented the process through which the receiver acquired the communication key and the hash value of the sender’s identity. The substitution transition Send_Teach-in represented when the sender constructed the teach-in message. The substitution transition Receive_Teach-in represented that the receiver decrypted teach-in messages from the sender. The substitution transition Send_Message represented that the sender submitted the authentication request. The substitution transition Send_Message’ represented the process of verifying the sender for the receiver. Figure 11 depicts the specific procedure.

**Figure 12:**
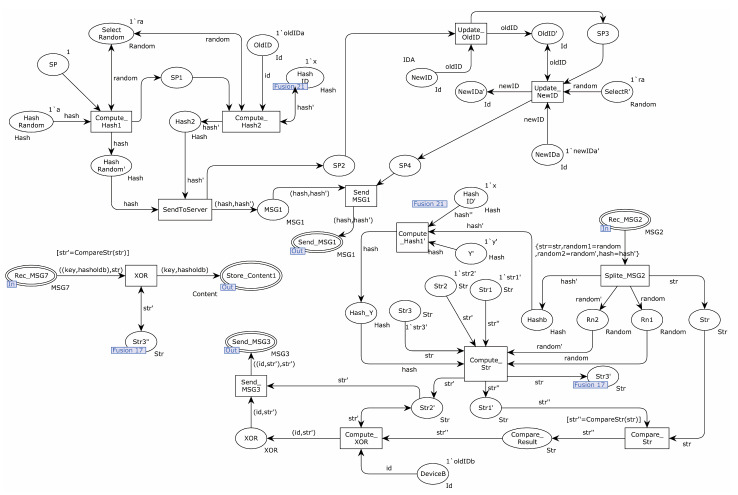
Substitution transition Authentication_Server internal model.

Figure 12 shows the detailed process for the substitution transition of Authentication_Server. There were four steps in this transition, which were: the sender sent the communication key request to the server, the sender received the authentication request from the server, the sender saved the communication key and hash value of the receiver’s identity from the server, and the sender updated the hash value of its identity.

The specific process of sending a communication key request from the sender to the server is shown below. The place SelectRandom sent a random number to the transition Compute_Hash1, and the transition Compute_Hash1 calculated the hash of this random number, sent the calculated hash to the transition SendToServer, and sent the random number to the transition Compute_Hash2. The hash′ was calculated using the random number and the identity ID, and the hash′ was sent to the transition SendToServer. Finally, the transition SendToServer sent the two hashes to the place Send_MSG1.

The specific process of updating the identity ID at the sending end was as follows. When MSG1 was sent successfully, the transition Update_OldID obtained the new identity ID of the device and sent the new identity ID to the transition Update_NewID. The transition Update_NewID saved the received identity ID to the place OldID′ and used the received identity ID and a random number to calculate the new identity ID of the device, saving the new identity ID to the place NewIDa′.

The specific process of receiving an identity request from the server at the sending end was as follows. Place Rec_MSG2 sent the message to the transition Split_MSG2. The transition Split_MSG2 decomposed the received message, sent str to the transition Compare_Str, and sent hash′, random′, and random to the transition Compute_ Str. The transition Compute_Hash1′ used the previously calculated hash′′ and the received hash′ to calculate the new hash and sent the hash to the transition Compute_Str. The transition Compute_Str used the random numbers random′ and random to decompose the hash into str, str′, and str′′ and saved str to the place str1′. str′′ was saved to place str2′, and str′′ was saved to place str3′. The transition Compare_Str compared str′′ with str. If different, the subsequent steps were stopped. If not, str′′ was sent to the transition Compute_XOR, which calculated the difference between id and XOR and sent it to the transition Send_MSG3.

The process of saving the communication key from the server and the receiver’s identity hash on the sending side was as follows. Place Rec_MSG7 sent the received message to the transition XOR, which used str′ to compute the heterogeneous value of the received message and sent the computation structure to the place Store_Content1.

**Figure 13:**
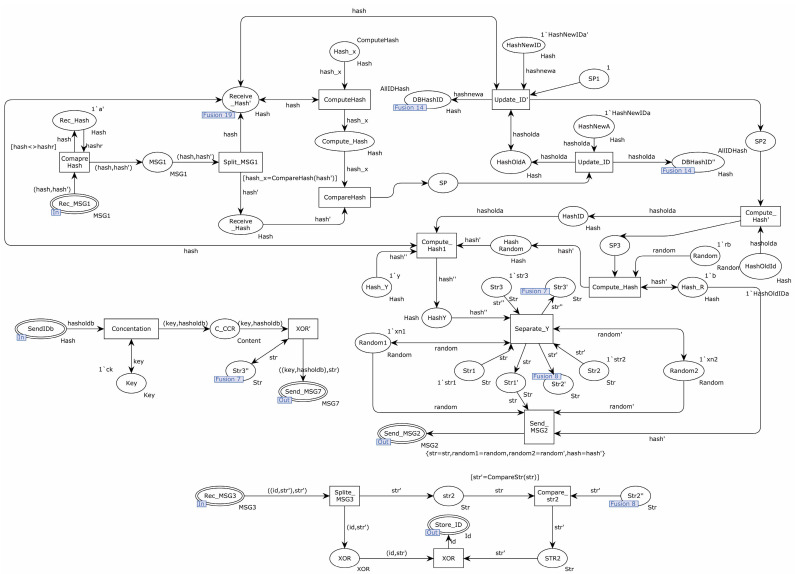
Substitution transition Authentication_A internal model.

Figure 13 shows the internal model of substitution Authentication_A. This substitution transition consisted of three specific processes: the server authenticated the sender, the server sent the authentication request to the sender, the server sent the authentication request to the receiver, and the server sent the communication key and the receiver’s identity hash value to the receiver.

The specific process of the authentication of the sender by the server was as follows. The place Rec_MSG1 sent the message to the transition Compare_Hash, which compared the hash in the message to prevent replay attacks. If a replay attack was detected, all the subsequent steps were terminated. Otherwise, the message was sent to the transition Split_MSG1. The transition Split_MSG1 split the message, sent the hash to the transition ComputeHash, and sent the hash′ to the transition CompareHash. The transition ComputeHash calculated the hash′ from the hash′. If hash_x was the same, the server authenticated the sender successfully, and the transition Update_ID′ and the transition Update_ID updated the hash value of the sender, saving the result to place DBHashID′.

The process of sending an authentication request from the server to the sender was as follows. The transition Compute_Hash calculated the hash of a random number and sent the calculated hash to the transition Compute_Hash1. The transition Compute_Hash1 calculated the hash, hasholda, and hash′ and sent the hash to the transition Separate_Y. The transition Separate_Y divided the incoming hash into str, str′, and str′′ strings. Finally, the transition Send_MSG2 sent str, two random numbers, and hash′ to the place Send_MSG2.

The exact process of sending an authentication request from the server to the receiver is shown below. Place Rec_MSG3 sent the message to the transition Split_MSG3. The transition Split_MSG3 split the received message and sent str to the transition Compare_str2. The transition Compare_str2 compared the received str with the str’ calculated by the server, and if it was the same, it meant the server successfully authenticated the receiver. The server sent the str to the XOR, which computed the message (id, str) and sent the result to the place store_ID.

The server sent the communication key and the receiver’s identity hash to the receiver as shown below. Place SendIDb sent the received message to the transition Concatenate, which sent the receiver’s identity hash and communication key to the transition XOR’. The transition XOR’ performed an XOR operation between the received message and the string str and sent the result to the place Send_MSG7.

### 6.2. EnOcean-A Protocol Security Assessment

**Figure 14:**
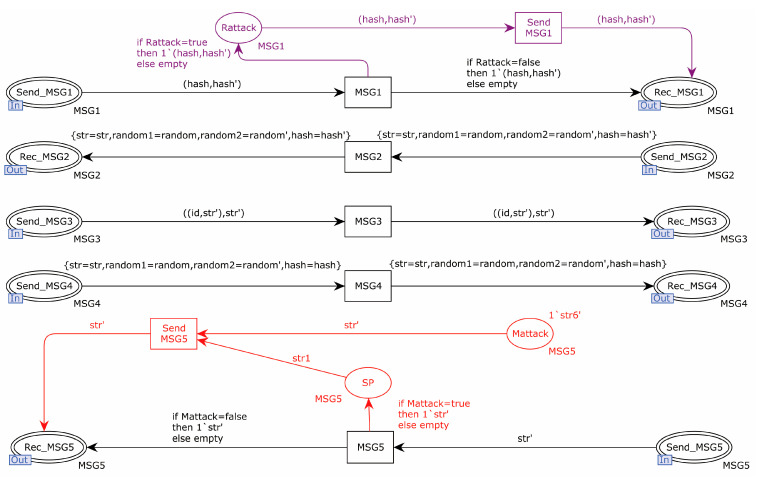
Tamper attack and replay attack at the network transport layer.

**Figure 15:**
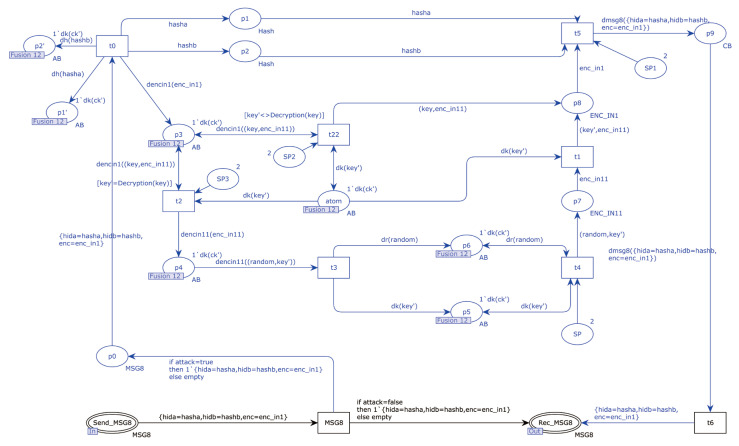
KCIA at the network transport layer.

The Dolev–Yao attacker model was introduced to attack the network level of the new scheme, including a tamper attack, replay attack, and KCIA. The purple part indicates the replay attack, the red part indicates the tamper attack, and the blue part indicates the KCIA. The details are shown in Figure 14 and Figure 15.

Table 7 compares the state-space report of the EnOcean-A protocol with the state-space report of the EnOcean-A protocol after adding the attack.

**Figure 16:**
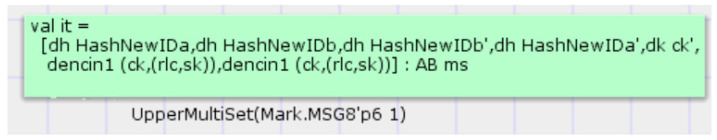
Information search in attacker’s place.

**Figure 17:**
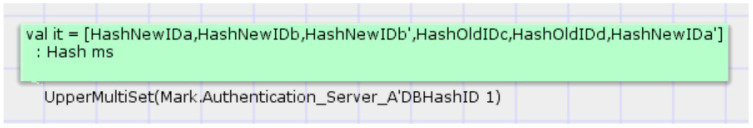
Information search in trusted third-party server’s place.

According to Table 7, the EnOcean-A protocol’s state space report shows that there was one dead node and zero dead transitions, indicating that the protocol could operate normally and that identity authentication between the sender and the receiver was successful. The EnOcean-A protocol added the tamper attack. A communication could be tampered with because the receiver delivered the authentication result in plain text. The 40 dead transitions were the result of the failed authentication, which the server detected when it checked the message and discovered that it did not match. The protocol added the replay attack since the sender created the information request while requesting the communication key from the server using a random number. The server assessed the message’s random number after receiving the sender’s communication key request for information. When the random number was found to be received, the key request failed due to instant determination that a replay attack occurred. The server identified a replay attack that resulted in 81 dead transitions. The KCIA was introduced in the protocol because the sender requested the communication key from the server before communicating with the receiver, so the communication key used by the communicating parties was different each time they communicated, and the four dead changes were due to the attacker’s inability to break the encrypted message. By querying the repository of the attacker’s intercepted content, it can be found that, since the hash value of the identity information was updated after the receiver and the recipient finish communicating with the server, a different hash value was used for each communication, so the attacker could not obtain the specific information of the communicating device, as shown in Figure 16. By querying the server’s place, it was found that the trusted third-party server updated the hash of the device’s identity information at each communication, as shown in Figure 17. Since the EnOcean-A protocol updated the communication key at each communication, the attacker could not decrypt the obtained information and could not know the detailed content of the message transmission. Thus, the attacker could not launch a tamper attack, replay attack, or KCIA on the EnOcean-A protocol. Moreover, the EnOcean protocol used a homomorphic hash function to anonymize the device identity, so the attacker could not determine whether a message was sent from a device.

### 6.3. EnOcean-A Security Analysis

In the following, the proposed scheme was secure against various known attacks.

- Replay attack: The attacker eavesdropped the transmitted data and resent the eavesdropped data to the receiver in the next round of inter-node communication to achieve the purpose of cheating the receiver. In the EnOcean-A protocol, the sender and receiver sent hash values that generated random numbers when communicating with the server, so when the device received a message, it first determined whether the random number hash value in the message already existed; if it did, it directly discarded the message. The teach-in message sent by the sender also included a random number. When the receiver received the message, it checked whether the random number was already received. If it was found that the random number was already received, then the message was discarded directly.
- Impersonation attack: The attacker used the intercepted device identity information to initiate a session request to other devices. In the EnOcean protocol, the attacker could not obtain the device’s identity launch impersonation attack because the device information was anonymized before communication, and the device anonymization information was updated at each communication.
- Tamper attack: The attacker intercepted the message transmitted using plaintext and sent the tampered message to the receiving end. In the EnOcean-A protocol, the sender and receiver used partial hashes of identity for transmission when communicating with the server. Even if an attacker intercepted and tampered with the hash value of the message, when the two communicating parties received the message, they compared it with the hash value of their own saved identity and discarded the message if they found that the hash value was different. In the authentication phase, the sender used the session key to encrypt the calculated CMAC value, and the attacker could not tamper with the message without knowing the session key.
- Eavesdropping attack: The attacker used a passive attack to eavesdrop on the data transmitted in the network and analyzed the data to launch an attack on the node. In the EnOcean-A protocol, since the sender and receiver sent partial hash values of the devices when communicating with the server, even if the attacker eavesdropped on the transmitted messages, they could not construct a complete hash of the device identity.
- KCIA: In the EnOcean-A protocol, the communication key was sent from the trusted third-party server to both communicating parties, and both communicating parties obtained the communication key from the trusted third-party server before each communication. Even if an attacker broke the communication key used during a certain communication, he could not use this communication key to decrypt the contents of messages transmitted before or after. The freshness of the communication key was also guaranteed. This scheme could provide backward and forward security.
- Anonymity: In the EnOcean-A protocol, the sender must obtain the hash of the receiver’s identity from the trusted third-party before communicating with the receiver, and when the sender communicated with the receiver, the sender also used the hash of the identity for message delivery. Therefore, the communication process could ensure the anonymity of device information.
- Unlinkability: In the EnOcean-A protocol, the sender used an updated hash each time it communicated with the trusted third-party, and the trusted third-party server also updated the hash of the receiver. Therefore, the attacker could not deduce the specific communication device from all the hash values eavesdropped after obtaining the communication information.

Due to the limited memory and performance of EnOcean device chips, the new protocol introduced a trusted third-party server to address the EnOcean protocol’s security issues. To guarantee that the protocol was resistant to a KCIA, the trusted third-party server produced and delivered the communication key to the communication parties. The EnOcean-A protocol used a homomorphic hash function that could be run securely on lightweight devices such as EnOcean. In addition, the EnOcean-A protocol could ensure that communication devices remained untraceable and anonymous. Table 8 compared the security analysis of EnOcean-A protocol with EnOcean protocol. The data in the table fully showed that EnOcean-A protocol has stronger security. 

## 7. Conclusions

This paper addressed the security problem that sensitive data transmitted by devices in intelligent lighting control systems can be easily obtained by malicious attackers and used to analyze the personal privacy of users based on intercepted messages. It took the EnOcean protocol as the research object. The formal analysis of the EnOcean protocol using CPN theory and the Dolev–Yao attacker model revealed that the EnOcean protocol was insecure against a KCIA, and the identity information of communication devices was not anonymized, so an attacker could analyze a user’s life status based on the eavesdropped device identity information. To address the above issues and due to the limited computing power and storage control of EnOcean devices, a EnOcean-A protocol was proposed. The improved protocol introduced a trusted third-party server to send communication keys, as well as each time the communication device needed to obtain a new communication key from the trusted third-party server, which was designed to resist a KCIA and provide forward security. The device identity information was dynamically updated so that the attacker could not analyze the user’s usage from the eavesdropped device identity information, ensuring the unlinkability of the device identity information. A formal analysis of the EnOcean-A protocol revealed that the improved protocol could effectively resist the security problems existing in the EnOcean protocol and provide stronger security. In this paper, we only focused on improving the security of the protocol and did not sufficiently consider the real-time aspect of the protocol communication. In future work, we plan to consider the protocol to improve the security and reduce the time cost to achieve real-time requirements as much as possible.

## Figures and Tables

**Table 1:** Comparison of related works.

	Main Contribution	Drawbacks
Sadri et al. [24]	Proposed a two-factor authentication protocol	These three authentication schemes required user’s physical information and were not applicable to device-to-device authentication protocol
Banerjee et al. [25]	Presented a three-factor authentication scheme	
Fakroon et al. [26]	Proposed a new authentication scheme that combined physical context	
Hajian et al. [27]	Suggested an authentication protocol between two devices	Neither of these protocols used a third-party server. The computational performance of lightweight sensor devices could not perform the key computation and complex encryption algorithms, so such designs were not suitable for communication between lightweight sensor devices with limited computational power and storage space
Li et al. [28]	Proposed an anonymous authentication and key negotiation protocol	
Hasan et al. [29]	Proposed a lightweight, secure smart home protocol based on mutual anonymous authentication and key negotiation of devices	These two schemes were only applicable for authentication between three points: the user, sensor node, and gateway node. They were not applicable to device-to-device authentication services
Banerjee et al. [30]	Suggested a more secure and robust authentication scheme	
Rasheed et al. [31]	Proposed a zero-knowledge proof-based authentication mechanism	This approach could be used with a multicast environment’s identity authentication protocol. It was not necessary to authenticate between two nodes
Shuai et al. [32]	Proposed an efficient, anonymous authentication scheme based on ECC for smart home environments	The ECC public key encryption scheme was used in this scheme, which has a certain demand on the performance of the device. However, the computational performance of lightweight sensor devices could not accomplish the key computation and the complex encryption algorithm
Kumar et al. [33]	Suggested an anonymous security framework for smart home environments	The device information in this scheme did not change dynamically and did not provide untraceability of communication devices

**Table 2:** Symbolic representation of EnOcean communication process.

Symbol	Definition
$ID_{A}$	Chip ID of sending device
$ID_{B}$	Chip ID of receiving device
A, B	Sender A, Receiver B
RLC	Rolling code
PSK	Pre-shared key
Key	Session key
RND	Random number
Payload	Payload of telegram
CMAC, CMAC’	Cipher-based message authentication code
$Enc\left(\cdot \right)$	Encryption function

**Table 3:** EnOcean protocol color set definitions.

Key Elements	Color Set Definition
ID	colset ID = with ida | idb;
RANDOM	colset RANDOM = with rlc | rndb | rndb’ | none;
KEY	colset KEY = with S | SA;
PAYLOAD	colset PAYLOAD = STRING;
MSG1	colset MSG1 = record ida:ID × idb:ID × enc:ENC_IN1;
MSG2	colset MSG2 = record ida:ID × idb:ID;
MSG3	colset MSG3 = record ida:ID × idb:ID × rnd:Random;
MSG4	colset MSG4 = record ida:ID × idb:ID × enc2:ENC2;

**Table 4:** State-space analysis of EnOcean protocol model.

Type	Number
State-Space Nodes	1380
State-Space Arcs	3582
Scc Graph Nodes	1380
Scc Graph Arcs	3582
Dead Marking	1
Dead Transition	0

**Table 5:** State space analysis of the model.

Type	Tamper Attack	Replay Attack	KCIA
State Space Nodes	7380	131	4537
State Space Arcs	24,672	280	12,318
Scc Graph Nodes	7380	131	4537
Scc Graph Arcs	24,672	280	12,318
Dead Markings	1	1	1
Dead Transitions	0	23	0

**Table 6:** Symbolic representation of EnOcean-A communication process.

Symbol	Definition
$OldID_{x}$	Old ID of device X
$NewID_{x}$	New ID of device X
$H\left(.\right)$	One-way hash function
$r_{i},\;n_{i}$	$i^{th}$ random number
$Str_{i}$	String
$Device_{i}$	Device name
RLC	Rolling data
p	Payload
SK	Session key
CK	Communication key
RND	Random number
$HID_{i}$	Hash value of device I’s ID
A, B, S	Device A, Device B, and Server S
$CMAC\left(.\right)$	Cipher-based message authentication code
⊕	XOR operation
∥	Concatenation operation
$Enc\left(\cdot \right)$	Encryption function

**Table 7:** State-space analysis of EnOcean-A protocol.

Type	EnOcean-A	Tamper Attack	Replay Attack	KCIA
State-Space Nodes	726	48	9	1326
State-Space Arcs	944	47	8	2224
Scc Graph Nodes	726	48	9	1326
Scc Graph Arcs	944	47	8	2224
Dead Markings	1	1	1	1
Dead Transitions	0	40	81	4

**Table 8:** Security comparison between EnOcean protocol and EnOcean-A protocol.

Protocol	Tamper Attack	Replay Attack	Impersonation Attack	KCIA	Anonymity	Unlinkability
EnOcean	×	×	√	√	√	√
EnOcean-A	×	×	×	×	×	×

## Data Availability

No data were used to support this study.
